# More than a photoreceptor: aureochromes are intrinsic to the diatom light-regulated transcriptional network

**DOI:** 10.1093/jxb/erae004

**Published:** 2024-03-27

**Authors:** Sacha N Coesel

**Affiliations:** School of Oceanography, University of Washington, Seattle, WA, USA

**Keywords:** Aureochromes, blue light photoreceptors, diatoms, phytoplankton, photosynthetic stramenopiles, gene regulation, protein–DNA binding

## Abstract

This article comments on:

Im SH, Lepetit B, Mosesso N, Shrestha S, Weiss L, Nymark M, Roellig R, Wilhelm C, Isono E, Kroth PG. 2024. Identification of promoter targets by Aureochrome 1a in the diatom *Phaeodactylum tricornutum*. Journal of Experimental Botany 75, 1834–1851.

**Aureochromes are light-responsive transcription factors, containing a blue light-sensitive light-, oxygen-, voltage-sensing (LOV) domain and a basic leucine zipper (bZIP) DNA-binding domain. Aureochrome photoreceptors are widespread in photosynthetic stramenopile algae important for aquatic ecosystems. Using targeted DNA and protein binding assays, Im *et al.* (2024) showed that aureochromes can form homo- and heterodimers, activating the expression of intermediate regulatory factors in the model diatom *Phaeodactylum tricornutum.* The use of mutant cell lines illustrated a light regulatory network in marine diatoms that is divergent from that of land plants.**

Aureochrome photoreceptors are a mainstay in the aquatic environment, expressed by a class of phytoplankton that are important for sustaining the aquatic food web (Box 1; Fig. 1). Aureochromes consist of a bZIP domain in the central region of the protein and a LOV domain at the C-terminus. This domain combination is not found in terrestrial plants or animals. The LOV domain can bind the UV/blue light-excitable FMN chromophore, and the protein functions as a blue light-regulated bZIP transcription factor (Takahashi *et al.*, 2007). Unlike other photoreceptors, aureochromes circumvent typical photoreceptor signal transduction phosphorylation cascades and can directly bind to target DNA upon blue light activation and dimerization (Hisatomi *et al.*, 2014; Nakatani and Hisatomi, 2015). This fast-acting light-controlled regulatory property has piqued the interest of the optogenetics community. For phytoplankton, this property may provide a competitive edge in aquatic systems that impose a uniquely dynamic light field to organisms being swept up and down the water column, exposing them to wide ranges in light intensity and spectral quality.

Box 1. Marine plankton communities have appreciable levels of organismal diversity, employing an array of different life strategies. About a decade ago, a consortium of scientists undertook the concerted effort to sequence, assemble, and annotate 650 transcriptomes of diverse marine protists as part of the Marine Microbial Eukaryote Transcriptome Sequencing Project (MMETSP), which propelled the field of marine biology forward. Within these MMETSP transcriptomes, aureochromes can be identified across the photosynthetic members of the stramenopile clade (Coesel *et al*., 2021), with the *Aureo2* paralog being restricted to diatoms. Aureochromes were also identified in two kleptoplastic dinoflagellates, sometimes called ‘dinotoms’ (*Alveolata*), indicating that their enslaved diatoms still expressed these regulators, and in the ciliate *Tiarina fusus* (*Ciliphora*) in co-culture with aureochrome-expressing prey.

**Fig. 1.**
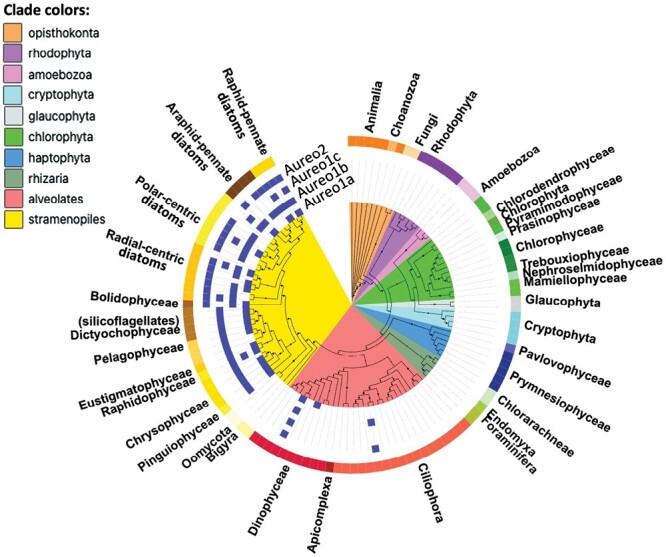
Taxonomic distribution of aureochromes in marine plankton. Presence/absence of aureochrome paralogs in 117 different eukaryotic orders relevant for the marine environment, visualized on an 18S rRNA maximum-likelihood phylogenetic tree (adapted from Coesel *et al*., 2021). The taxonomic phylum- and class-level classifications are indicated by the colored ranges.

## Aureochrome-utilizing organisms are photosynthetic, yet not related to plants

Aureochromes were first identified and characterized in the filamentous yellow-green alga *Vaucheria frigida* (*Xanthophyceae*) and the brown seaweed *Fucus distichus* (*Phaeophyceae*) (Takahashi *et al.*, 2007). In these photosynthetic organisms, aureochromes were found to regulate photomorphogenic processes such as the formation of branches and sex organs (Takahashi *et al.*, 2007). Aureochromes were thereupon identified across different members of the photosynthetic stramenopile algal clade (Box 1; Fig. 1). The evolutionary history of stramenopiles is different from that of green algae and land plants. The stramenopile plastid arose from secondary endosymbiosis involving a heterotrophic protist and a primitive red alga (Delwiche, 1999). As a result, there are significant structural differences in stramenopile chloroplast and thylakoid structures compared with the green lineage, including different (brown) accessory pigments for light harvesting. Some motile photosynthetic stramenopiles have the capacity to supplement nutrition by phagocytotic feeding (Adl *et al.*, 2012). In addition to aureochromes, stramenopile protists evolved other unique light-sensing proteins, such as dual-functioning cryptochromes (Coesel *et al.*, 2009), phytochromes (Fortunato *et al.*, 2016), microbial rhodopsins (Govorunova *et al.*, 2017), helmchromes (Regulator of G protein signaling; RGS-LOV proteins) (Fu *et al.*, 2015), and other variations of potential light-sensitive proteins that show distinctive diel expression profiles in *in situ* plankton communities, but whose functions are as yet unknown (Coesel *et al.*, 2021).

## Aureochromes in *Phaeodactylum tricornutum*, a preferred diatom model organism

Most of the molecular work published on aureochromes was done in the diatom *P. tricornutum*. This organism has been used as a model for over three decades, aided by a stable genetic transformation method established in 1999 (Falciatore *et al.*, 1999), and by its sequenced genome ~10 years later (Bowler *et al.*, 2008). Diatoms are unicellular, photoautotrophic stramenopiles encapsulated by a silicified cell wall. They are a highly successful branch in the aquatic environment, comprising over an estimated 100 000 species (Adl *et al.*, 2012). The *P. tricornutum* genome encodes four different aureochrome paralogs: *PtAureo1a*, *PtAureo1b*, *PtAureo1c*, and *PtAureo2* (Schellenberger Costa *et al.*, 2013). These genes exhibit temporal differences in expression, suggesting distinct functions. In *P. tricornutum*, *PtAureo1a* and *1c* are diurnally regulated, while the expression of *PtAureo1b* is light induced. *PtAureo2* cannot bind the chromophore FMN due to steric hindrance in its structure caused by a point mutation (Banerjee *et al.*, 2016a) and shows constitutive expression under laboratory conditions (Banerjee *et al.*, 2016b). A pioneering study indicated that PtAUREO1a may be involved in the light-dependent cell cycle regulation of *P. tricornutum* by activating the diatom-specific cyclin 2 (*dsCYC2*) promoter in response to light (Huysman *et al.*, 2013). RNAi-silenced ptAUREO1a cell lines furthermore indicated a role for this protein in photosynthetic acclimation to light (Schellenberger Costa *et al.*, 2013), and *ptAureo1a* knockout (KO) lines showed a severe dysregulation of gene expression levels in blue and red light conditions (Mann *et al.*, 2020), indicating a pivotal role for PtAUREO1a in gene regulation.

## It’s all about dimerization

**Fig. 2.**
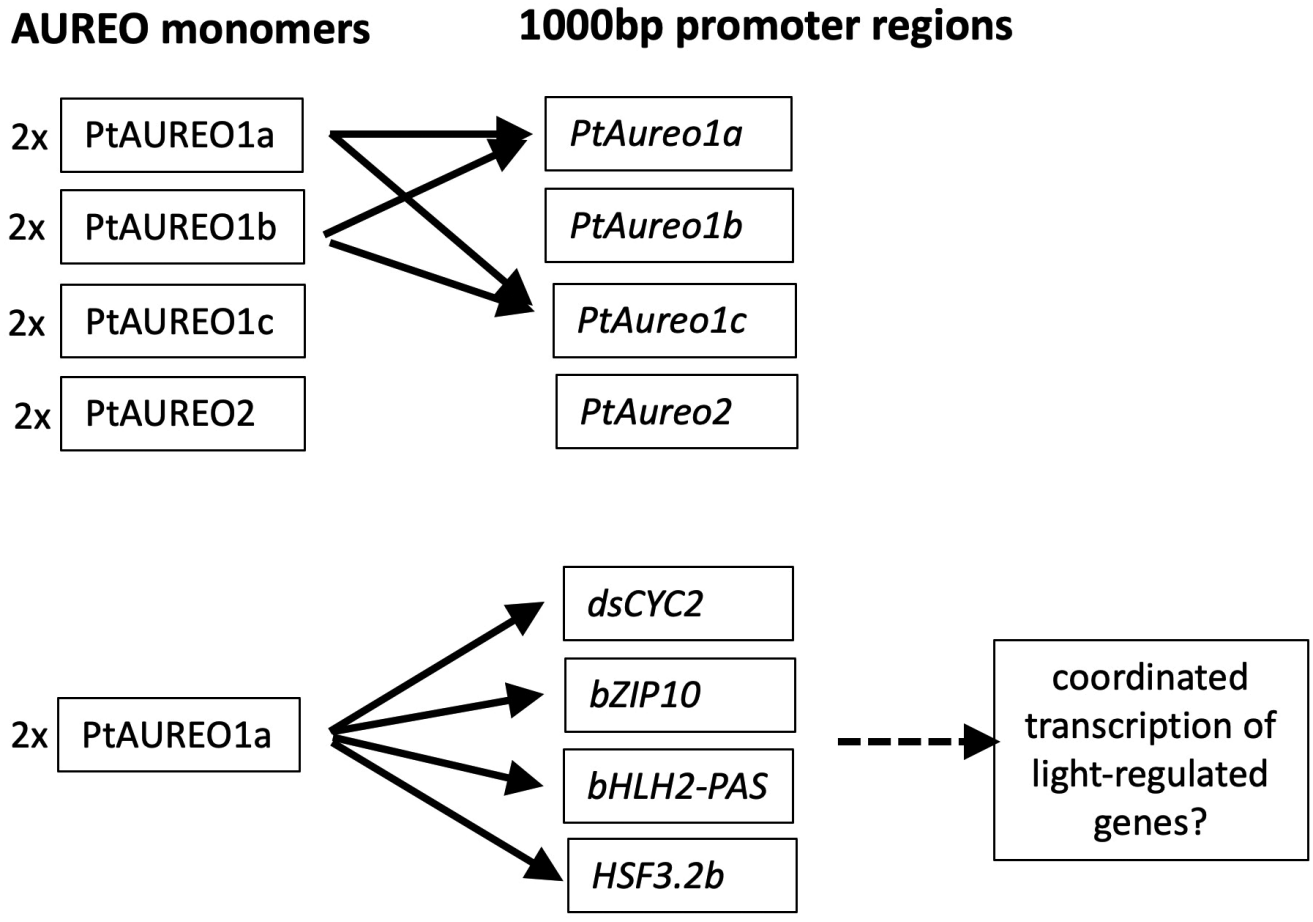
Promoter binding and transcriptional activation by aureochrome homodimers. Schematic representation of the results presented by Im *et al.* (2024).

The bZIP transcription factors must form dimers to bind their target DNA. It was quickly understood that PtAUREO1a can form homodimers, as well as heterodimers with PtAUREO1c and PtbZIP10 (Huysman *et al.*, 2013; Banerjee *et al.*, 2016b), and that blue light induces conformational changes of PtAUREO1a (Hisatomi *et al.*, 2014; Nakatani and Hisatomi, 2015; Banerjee *et al.*, 2016b). Im *et al.* (2024) took this work further with a systematic approach for identifying new dimerization partners of the four aureochrome proteins. Using *in vitro* pull-down assays on glutathione *S*-transferase (GST)–PtAUREO fusion proteins, the authors demonstrated that all four PtAUREO paralogs can form dimer partners with themselves and with each other, including the PtAUREO2 protein that does not bind an FMN chromophore. Most of these pull-down assays were successful in both light and dark conditions. The resulting 10 different combinatorial partners may have different DNA binding specificity that could potentially broaden their regulatory targets *in vivo*. Im *et al.* (2024) further explored the DNA binding properties of each of the four PtAUREO homodimers using a heterologous yeast one-hybrid (Y1H) system. They tested DNA binding and transcription initiation using each individual aureochome promoter region as bait and found that homodimers of both PtAUREO1a and PtAUREO1b interact with the promoter sites of *PtAureo1a* and *PtAureo1c* genes in both dark and blue light conditions (Box 2; Fig. 2). These results suggest a self-regulatory feedback system that may be independent of the light environment, at least within this heterologous system. PtAUREO1c and PtAUREO2 showed little to no measurable interaction with any of the four aureochrome promoters, suggesting that these aureochromes may have other DNA targets, or that they play a different role in the cell, for example in heterodimerization.

Box 2. Using yeast one-hybrid interaction assays, Im *et al*. (2024) demonstrated that PtAUREO1a and 1b proteins can bind and activate the promoter regions of *PtAureo1a* and *1c*. PtAUREO1a was furthermore shown to interact with the promoter regions of other transcription factors and the cell cycle regulator dsCYC2. The work of Im *et al*. (2024) illustrates that the four aureochromes of *P. tricornutum* are co-operating partners and suggests that PtAUREO1a regulates the expression of intermediate transcription factors that control the light-regulated transcriptome of *P. tricornutum.*

## Resilience in light responses

While *PtAureo1a*-KO lines have strongly affected expression profiles upon blue light illumination (Mann *et al.*, 2020), Im *et al.* (2024) showed that the PtAUREO1a protein itself interacted only with a small subset of tested target genes. Most of these genes encode transcription factors, namely bZIP10, bHLH2-PAS, PtHSF3.2b, PtAUREO1a, and PtAUREO1c. These results suggest that PtAUREO1a is part of a light-sensitive gene-regulatory network of *P. tricornutum*, rather than acting as a direct transcription factor to a wide array of light-responsive target genes. In addition, long-term red light acclimation also led to phenotypic and gene expression phenotypes in *PtAureo1a*-KO cell lines (Schellenberger Costa *et al.*, 2013; Mann *et al.*, 2020), which may further support the idea of PtAUREO1a as part of an intricate light regulatory network encompassing multiple light sensing and regulatory modules beyond blue light regulation. To address this, Im *et al.* (2024) reconstituted a *PtAureo1a*-KO cell line with a mutated aureochrome gene with reduced FMN binding properties. This mutated protein, PtBLINDA1a, showed altered aureochrome promoter binding properties in Y1H assays, and the BlindA1a mutants showed a shift in PSII fluorescence emission spectra in red light-acclimated cells caused by a down-regulation of expression of the light-harvesting protein gene *Lhcf15*. The authors also demonstrated that PtAUREO1a did not directly interact with the promoter of the *Lhcf15* gene, thus again suggesting a role for intermediate regulatory partners. Im *et al.* (2024) further showed that most wild-type phenotypes in both red and blue light conditions were recovered in the BlindA1a mutants, indicative of a general resilience of the mutant light responses despite modifications to one of its light-sensing components. The other aureochrome paralogs, as well as the light-sensitive cryptochromes (Coesel *et al.*, 2009) and phytochromes (Fortunato *et al.*, 2016) and their intermediate partners, could potentially contribute to the resilience in light sensing and regulation that is important for a photosynthetic organism residing in a naturally turbulent environment.

## Going forward

As shown by Im *et al.* (2024) and in previous studies, aureochromes are important regulators of gene expression. Studies so far have been using targeted approaches on select candidates to identify dimerization partners and DNA binding interactions. These test candidates were mainly chosen based on experimental results with *PtAureo1a*-KO cell lines grown under very specific light conditions. While targeted approaches are powerful, they can also lead to a narrow view in which novel and unexpected interacting partners are overlooked. This may be especially true when dealing with complex regulatory networks.

One way to expand our knowledge on the regulatory network components in which aureochrome is involved is to combine bioinformatics approaches with high-throughput sequencing to identify novel interacting factors in the *P. tricornutum* genome and proteome. In the diatom *Thalassiosira pseudonana*, the combination of transcriptional expression profiling with upstream DNA-binding promoter analysis led to the identification of TpAUREO1c as a potential regulator of dawn-regulated genes in exponentially growing cells (Ashworth *et al.*, 2013). Adding high-throughput sequencing approaches, such as *in vitro* DNA affinity purification sequencing (DAP-seq) or ChIP sequencing (ChIP-seq), would greatly improve our confidence in such inferred gene-regulatory network analyses. Likewise, novel aureochrome protein interaction partners may be identified using co-immunoprecipitation techniques, broadening our understanding of the light regulatory network of this alga.

Furthermore, the studies on aureochromes have focused on a limited number of species that are primarily model organisms. In the case of diatoms, *P. tricornutum* is a versatile study subject, yet not representative for the dominant diatom species found in the world oceans. Recent studies on *Nannochloropsis oceanica* (Poliner *et al.*, 2022) and the brown seaweed *Saccharina japonica* (sweet kelp) (Wu *et al.*, 2022) are starting to address this, but major stramenopile lineages with diverse lifestyles have yet to be investigated. The recent expansions in molecular tools and sequencing resources for ecologically important stramenopile species will allow us to compare the light regulatory machineries of diatoms and other stramenopiles evolved in a diversity of light fields, such as the polar diatom *Fragilariopsis cylindrus* that is subjected to prolonged periods of darkness. The work provided by Im *et al*. (2024) is leading the way towards more exciting research to come.
